# Extracellular Vesicles Secreted by *Neospora caninum* Are Recognized by Toll-Like Receptor 2 and Modulate Host Cell Innate Immunity Through the MAPK Signaling Pathway

**DOI:** 10.3389/fimmu.2018.01633

**Published:** 2018-07-24

**Authors:** Shan Li, Pengtao Gong, Lixin Tai, Xin Li, Xiaocen Wang, Chunyan Zhao, Xu Zhang, Zhengtao Yang, Ju Yang, Jianhua Li, Xichen Zhang

**Affiliations:** College of Veterinary Medicine, Jilin University, Changchun, China

**Keywords:** *Neospora caninum*, extracellular vesicles, innate immunity, toll-like receptor 2, MAPK

## Abstract

*Neospora caninum* is an obligate intracellular parasite, which causes significant economic losses in the cattle industry. However, the immune mechanism of the parasite–host interaction is not yet fully understood. Extracellular vesicles (EVs) have emerged as a ubiquitous mechanism by which almost all cells, especially immune and tumor cells, participate in intercellular communications. Although studies have indicated that EVs secreted by *Toxoplasma gondii* or *Trypanosoma brucei* promote exchanges of biological molecules important for the host–parasite interplay, however, EVs and their biological activities in *N. caninum* is not clear. Here, we used multiple methods, including electron microscopy, nanoparticle tracking analysis, RT-PCR, immunofluorescence, western blot, proteomics, and cytokine analyses, to examine the properties of *N. caninum* EVs. We found that *N. caninum* produced EVs that are similar to mammalian exosomes, which generally range from 30 to 150 nm in diameter. It was shown that *N. caninum* EVs could remarkably increase the production of pro-inflammatory cytokines IL-12p40, TNF-α, IL-1β, IL-6, and IFN-γ by wild-type (WT) mouse bone marrow-derived macrophages (BMDMs) whereas the secretion of IL-12p40, TNF-α, and IFN-γ was very strongly downregulated in TLR2^−/−^ mouse BMDMs. The levels of IL-6 were not affected, but the secretion of IL-10 was upregulated. We found that the phosphorylation levels of P38, ERK, and JNK were significantly reduced in the TLR2^−/−^ cells compared with those in WT mouse BMDMs and that treatment with chemical inhibiters of P38, ERK, and JNK resulted in upregulation of IL-6, IL-12p40, and IL-10 production. Together, these results demonstrated that *N. caninum* EVs could be rapidly internalized to deliver proteins to the host cells and modulate the host cell immune responses through MAPK signaling pathway in a TLR2-dependent manner. Our study is the first to reveal potential roles for *N. caninum* EVs in host communication and immune response in parasite–host interactions.

## Introduction

*Neospora caninum* is an obligate intracellular parasite which naturally takes dog and cattle as hosts (1). Although *N. caninum* infectivity in humans has not been identified, serological evidence suggests that humans can be exposed to *N. caninum* (2, 3). The definitive hosts of *N. caninum* are mostly in the canid family, and infection mainly causes a variety of neurological symptoms, especially in dogs (4). Neosporosis is among the major causes of abortion in bovine. The calves vertically infected with the *N. caninum* show neurological disorders, which is costly for cattle farms and the related industry (5, 6). Global economic loss due to neosporosis is substantial (7, 8).

Similar to most other apicomplexan parasites, the life cycle of *N. caninum* can be broadly divided into three stages: tachyzoites, bradyzoites, and sporozoites (9). According to the literature, the pathogenesis of neosporosis is mainly caused by massive proliferation of *N. caninum* tachyzoites. Host innate immunity is a major player against protozoal infections *via* inhibiting parasite replication and triggering appropriate adaptive immune responses, which controls the active infections and overcomes subsequent re-exposures (6, 10). The recognition of highly conserved sets of molecular patterns [pathogen-associated molecular patterns (PAMPs)] by pattern-recognition receptors (PRRs) of host cells leads to the production of inflammatory cytokines (11). Previous studies have shown that the toll-like receptor 2 (TLR2), TLR3, and TLR11 innate recognition pathway triggers an inflammatory response to control *N. caninum* infection (12). However, the interaction of PAMPs in *N. caninum* with the PRRs on innate cells has not yet been fully elucidated, and the mechanisms by which antigens are presented to host cells are still unclear.

To ensure long-term survival in their host cells, parasites use diverse mechanisms, including extracellular vesicles (EVs) (13). EVs are produced by most cells which exhibit diverse functions and are ubiquitously involved in host-pathogen interaction (14). One recently described mechanism for release of proteins or lipids as well as infectious agents from parasites is through EVs. EVs have been shown to be important in communication and genetic exchange among microbes (15), and the functional research in parasites is still emerging. There are at least two major classes of EVs: exosomes, which are endocytic vesicles approximately 40–100 nm in size, and microvesicles, which are derived from shedding of the plasma membrane (16). Several protozoan parasites, including *Leishmania* species and *Trypanosoma brucei*, have been shown to release exosomes and/or microvesicles to regulate the host’s immune response. EVs from pathogens facilitate host immune evasion (17). Research has shown that *Leishmania* transports the GP63 protease to hepatic cells *via* exosomes to affect the parasite burden (18, 19). Moreover, *Leishmania* exosomes could trigger the release of anti-inflammatory cytokine IL-10 and inhibit the production of inflammatory cytokine TNF in human monocyte-derived dendritic cells (DCs) in response to IFN-γ (20). Mice inoculated with parasite microvesicles followed with infection with *T. cruzi* develop exacerbated cardiac manifestation and increased inflammatory infiltrates with elevated levels of IL-4 and IL-10 (21), suggesting that EVs from parasites participate in exchanges of information and genetic material between parasites and host cells. However, the characterization of *N. caninum* EVs and their biological activities have not yet been reported.

In the present report, we characterized the physical properties of EVs released by *N. caninum* and defined EVs-associated proteins. These EVs could be rapidly internalized thereby delivering their contents to the host cells. Furthermore, it was demonstrated that these *N. caninum* EVs could activate TLR2 in mouse bone marrow-derived macrophages (BMDMs) and that they had regulatory effects on the secretion of inflammatory cytokines through the TLR2 and MAPK signaling pathway, which indicated a potential role in parasite–host interactions.

## Materials and Methods

### Animals

Female C57BL/6 mice (6–8 weeks old) were purchased from the Changsheng Experimental Animal Center (Changchun, China), and TLR2-deficient (TLR2^−/−^) mice were obtained from the Model Animal Research Center of Nanjing University (Nanjing, China). All mice were housed under specific-pathogen-free conditions in the National Experimental Teaching Demonstration Center of Jilin University (Changchun, China). The food and water provided were sterile.

### Parasites and Cell Culture

*Neospora caninum* tachyzoites (Nc-1) were maintained by serial passage in VERO cells in RPMI-1640 medium, supplemented with 2% fetal bovine serum (FBS) (Biological Industries, Ltd., Kibbutz Beit-Haemek, Israel), 2 mM l-glutamine, 100 U penicillin, and 100 µg of streptomycin (P/S) (all from Life Technologies, Carlsbad, CA, USA) in a 5% CO_2_ atmosphere at 37°C. Free *N. caninum* were obtained from the cell cultures as described previously (22). Briefly, when 90% of the parasites were released from the host cell monolayer, the suspension was subjected to gradient density centrifugation in a 40% Percoll solution (v/v) (GE Healthcare, Uppsala, Sweden) at 1,500 × *g* for 30 min to remove the host cell debris. The parasite-containing pellet was collected and washed twice with PBS without P/S by centrifugation at 500 × *g* for 10 min. Approximately 5 × 10^8^ tachyzoites/ml were incubated for 24 h at 37°C in 5% CO_2_ in RPMI-1640 supplemented with 2% Exo-FBS (System Biosciences, CA, USA) but without P/S.

Bone marrow-derived macrophages were isolated using the previously described protocols (23). Briefly, 6- to 8-week-old wild-type (WT) C57BL/6 and TLR2^−/−^ mice were euthanized and immersed in 75% ethanol; the femurs and tibiae were removed, briefly sterilized in 75% ethanol, immersed in PBS + P/S for 5 min, and placed in RPMI-1640 + P/S. This medium was used to wash out the marrow cavity plugs, and the bone marrow cells were resuspended in 1640 supplemented with 25% L929-cell conditioned medium as a source of granulocyte/macrophage colony-stimulating factor with 10% FBS, 100 U/ml penicillin, and 100 µg/ml streptomycin, plated onto 100 mm dishes, and cultured at 37°C in a 5% CO_2_ atmosphere. The culture medium was changed 4 days post isolation. After 7 days, the adherent cells were removed and analyzed by flow cytometry. The positive phenotype was confirmed on the basis of having CD11b expression over 95%, and the cells were retained for further experimental procedures.

### *N. caninum* EVs and Antigen Preparation

*Neospora caninum* EVs were purified as previously described with a slight modification to the protocol (24). After 24 h in exosome-depleted medium, the parasite culture supernatant was collected and centrifuged at 300 × *g* for 10 min at 4°C to remove the parasites and then at 2,000 × *g* for 10 min to remove the debris. Finally, the resulting supernatant was centrifuged at 10,000 × *g* for 45 min. The supernatant was then passed through a 0.22-µm syringe filter (Millipore, Billerica, MA, USA), followed by further ultracentrifugation (Hitachi Micro Ultracentrifuge, Japan) at 100,000 × *g* for 70 min at 4°C to spin down the expected *N. caninum* EVs, which were then resuspended in 7 ml PBS. The EVs-rich fraction was washed twice with PBS, then resuspended in 200 µl PBS for further processing.

*Neospora caninum* lysate antigen (NLA) and excretory secretory antigens (ESAs) were prepared as previously described with a slight modification (25). For the preparation of NLA, approximately 1 × 10^9^ free *N. caninum* tachyzoites were resuspended in BAG buffer (116 mM NaCl, 5.4 mM KCl, 0.8 mM MgSO_4_, 50 mM Hepes, 5.5 mM d-glucose, pH 7.3) with protease inhibitors (KeyGen Biotech, Nanjing, China) and subjected to ultrasound (60 H/30 s) on ice. After centrifugation at 10,000 × *g* for 30 min at 4°C, the NLA was collected and filtered using 0.22 µm membranes. For the preparation of ESAs, approximately 2 × 10^8^ tachyzoites/ml were incubated at 37°C for 3 h in 2 ml serum-free RPMI-1640 containing P/S. After centrifugation for 10 min at 1,000 × *g*, the ESA-containing supernatant was filtered and collected for further processing.

The collected proteins were either stored at −80°C or directly used in additional experiments. The pellets were resuspended in protein loading buffer and stored at −20°C or immediately analyzed by western blot. All protein concentrations were measured using the BCA Protein Assay Kit (Thermo Scientific, Waltham, MA, USA).

### LAL Assay

To assessed the level of endotoxin in *N. caninum* EVs, NLA, and ESAs, the ToxinSensor™ chromogenic LAL Endotoxin Assay Kit (GenScript, Piscataway, NJ, USA) was used according to the manufacturer’s instructions. Briefly, 100 µl of standards, samples, and LAL reagent water were carefully dispensed into different endotoxin-free vials, then 100 µl LAL lysate was added and mixed well by swirling gently, incubated at 37°C for 30 min. After proper incubation, 100 µl of chromogenic substrate was added to each of the reactions and incubated at 37°C for 6 min. The reactions were terminated by adding 500 µl each of the three color stabilizer solutions and gently swirled to mix well. The endotoxin levels were then measured by spectrophotometry at 545 nm. Distilled water was used as blank to adjust the photometer to zero absorbance.

### PKH67-Labeled *N. caninum* EVs and Immunofrescence Staining

To examine whether *N. caninum* EVs can enter into BMDM, *N. caninum* EVs were labeled with the PKH67 Green Fluorescent Cell Linker Kit (Sigma, St. Louis, MO, USA) according to the manufacturer’s protocol with minor modifications. Fifty micrograms of *N. caninum* EVs were diluted in 100 µl PBS and added to 1 ml Diluent C, then 4 µl of PKH67 dye was added and incubated for 4 min in darkness, after which 1 ml 1% BSA/PBS was added to bind excess dye. The labeled EVs were washed at 100,000 × *g* for 1 h, and the pellet was diluted in 100 µl PBS in an opaque tube at 4°C and used for the internalization experiments. PBS was treated in parallel. An aliquot of 25 µg PKH67-labeled *N. caninum* EVs was added to BMDMs growing on glass coverslips in 24-well plates. At 0, 2, 4, 6, 8, and 12 h after treatment, the cells were washed twice with PBS, then dried at room temperature (RT) and fixed in 4% paraformaldehyde for 10 min, permeabilized with 0.25% Triton X-100 in PBS for 10 min and washed. F-actin was stained with TRITC Phalloidin (Yeasen, Shanghai, China), and the nuclei were stained with DAPI (Invitrogen, Carlsbad, CA, USA). We utilized *N. caninum* polyclonal antibodies against 14-3-3, HSP70, and enolase for confocal microscopy. The mouse polyclonal antibodies against Nc14-3-3, NcHSP70, and NcEnolase were prepared according to previously described protocols (26). Briefly, Nc14-3-3/HSP70/enolase were amplified from cDNA using PCR and cloned into the pEGX-4T-1 vector. The recombinant proteins were purified with Proteinlso^®^ GST Resin (TransGen Biotech, Beijing, China) according to the manufacturer’s protocol. Ten 8-week-old BALB/C mice were subcutaneously immunized with the obtained proteins, which were emulsified with Freund’s complete/incomplete adjuvant (Sigma, St. Louis, MO, USA), and finally the corresponding mouse polyclonal antibodies were generated. For immunolocalization, the protocol was similar to the method described above except that *N. caninum* EVs were not labeled, and at 3 and 6 h after treatment, the cells were washed twice with PBS, then dried at RT and fixed in 4% paraformaldehyde for 10 min. They were then permeabilized with 0.25% Triton X-100 in PBS for 10 min, blocked with 3% BSA in PBS (PBS-BSA) for 2 h at RT, and incubated with a 1:100 dilution of the 14-3-3, HSP70, or enolase antibody overnight at 4°C. Finally, they were washed and incubated with Alexa Fluor-conjugated secondary antibody (Proteintech, Wuhan, China) for 1 h at RT. The coverslips were mounted using DAPI before analysis on an Olympus FV1000 Laser Scanning Confocal microscope (Japan).

### RNA Extraction and Real-Time PCR Analysis

On day 7 after isolation, 3 × 10^6^ BMDMs were seeded in 6-well culture plates and incubated for 12 h with 50 µg/ml purified *N. caninum* EVs or Pam3Cys-Ser-(Lys) 4 (Pam3CSK4) (TLR2/TLR1 Agonist, Invitrogen, Carlsbad, CA, USA, 10 µg/ml) (27). RNA was extracted using TRIzol reagent (Invitrogen, Carlsbad, CA, USA) as previously described (28). All RNA precipitates were dissolved in 20 µl of nuclease-free water, and the RNA concentration and integrity were determined using a NanoDrop 2000 instrument (Thermo Scientific, Waltham, MA, USA). The cDNA synthesis was performed using 2 µg of total RNA in a final volume of 20 µl using a PrimeScript™ RT Reagent Kit (TaKaRa, Dalian, China) according to the manufacturer’s instructions. Real-time PCR was conducted using the qTOWER 2.2 system (Analytik Jena, Biometra) with the following steps: 95°C for 10 min; followed by 40 cycles of 95°C for 30 s, 58°C for 30 s, and 72°C for 30 s; and finally 72°C for 10 min. The data were normalized to GAPDH. All primers were synthesized by Kumei (Changchun, China), and their sequences were as follows: GAPDH, Forward: 5′-CCATGTTTGTGATGGGTGTG-3′, Reverse: 5′-CCTTCTTGATGTCATCATAC-3′; TLR2, Forward: 5′-CGCTCCAGGTCTTTCACCTC-3′, Reverse: 5′-AGGTCACCATGGCCAATGTA-3′. The relative expression levels of TLR2 were calculated using the 2^−ΔΔCt^ formula, where ΔΔCt represents the Ct (sample)-Ct (control). Every sample was tested in triplicate, and the data used for the final analysis were from three independent experiments.

### Transmission and Scanning Electron Microscopy

The enriched *N. caninum* EVs were resuspended in PBS and applied to a carbon-coated copper grid and incubated for 1 min at RT. After removal of the liquid using filter paper, 20 µl of 3% phosphotungstic acid was used to negatively stain the grid for 5 min at RT. The grid was dried for 15 min and then examined using transmission electron microscopy (HITACHI, Japan). For ultrastructural analysis of the secreted EVs, *N. caninum* tachyzoites were harvested after 6 h incubation. The pellet was then fixed with 2.5% glutaraldehyde in 0.1 M sodium cacodylate buffer overnight. After washing with the same buffer, the samples were post-fixed with osmium tetroxide and dehydrated in a graded acetone series prior to embedding in epoxy resin. Ultrathin sections (70–80 nm) were cut from the resin blocks using a Reichert-Jung Ultracut E Ultramicrotome. Formvar grids covered with isolated vesicles or with ultrathin sections were examined using TEM.

For SEM, *N. caninum* tachyzoites were cultured in 24-well plates (Corning, NY, USA) containing glass cover slips that had been pre-coated with 0.1 mg/ml poly-l-lysine (Sigma, St. Louis, MO, USA) and washed three times with distilled water. After a 6-h incubation, the cells were fixed with 4% glutaraldehyde for 24 h, washed with PBS and post-fixed in 1.0% osmium tetroxide. Then, the samples were dehydrated with a series of 30, 50, 70, 80, 90, 95, and 100% alcohol solutions, critical-point dried, sputter-coated with gold, and visualized with a Hitachi S-3400N scanning electron microscope (HITACHI, Japan).

### Nanoparticle Tracking Analysis

The *N. caninum* EVs were characterized for size distribution and quantitated using a ZetaView PMX 110 instrument (Particle Metrix, Meerbusch, Germany) and its corresponding software, ZetaView 8.02.28.

### LC-MS/MS Data Analysis

Liquid chromatography-tandem mass spectrometry (LC-MS/MS) was performed at Shanghai Omicsspace Biotech Co., Ltd. (Shanghai, China). Briefly, the proteins were precipitated with 15% trichloroacetic acid/acetone and digested with trypsin at a final concentration of 2 ng/ml. After incubation at 37°C for 18 h, the reactions were quenched by the addition of formic acid to a final concentration of 1% prior to the LC-MS/MS analysis. The buffers used for chromatography were 0.2% formic acid (buffer A) and 100% acetonitrile/0.2% formic acid (buffer B). The Thermo Scientific analytical column was equilibrated with buffer A before the samples were loaded from the autosampler to the EASY trap column (Thermo Scientific, EASY-nLC™1000) and then separated. The peptides were loaded onto the column and eluted with a three-slope gradient. Buffer B first increased from 5 to 28% in 40 min and then from 28 to 90% in 2 min, then remained at 90% for 18 min. The digested product was analyzed using an Orbitrap-Elite mass spectrometer (Thermo Finnigan, San Jose, CA, USA) after separation using capillary HPLC and desalting. The analysis time was 60 min.

### Protein Database Search and Bioinformatic Analyses

The MS/MS data were searched against the NCBI database with the Mascot software version 2.3 (Matrix Science). Mascot was set up to search the *N. caninum* database (21,511 sequences) with 20 ppm peptide mass tolerance and 0.1 Da fragment mass tolerance with allowance for two missed cleavages in the trypsin digests. Carbamidomethylation was specified in both search engines as a fixed modification, and oxidation of methionine residues was specified in Mascot as a variable modification. The identification of a protein was accepted if its peptides had FDR ≤0.01 (high confidence) and it contained at least two identified peptides. Annotations with gene ontology and Kyoto Encyclopedia of Genes and Genomes for identified proteins were also performed.

### Western Blot

The *N. caninum* protein was prepared as follows: free *N. caninum* tachyzoites were resuspended in CelLytic™ M lysis reagent with Benzonase (all from Sigma, St. Louis, MO, USA) and incubated at RT for 5 min; then, an equal volume of a solution of 2% SDS and 1 mM EDTA was added. For *N. caninum* EVs, the pellets were resuspended in ice-cold cell lysis buffer supplemented with a proteinase inhibitor mixture and a phosphatase inhibitor (BosterBio, Wuhan, China). Thirty micrograms of each protein sample were separated on a 12% SDS-PAGE gel, and the proteins were then transferred to PVDF membranes (Millipore, Bedford, MA, USA). After a 2-h blocking step in TBS-0.05% Tween 20 (TBST) containing 5% skim milk, the membranes were incubated at 4°C overnight with the following antibodies: polyclonal mouse antibodies anti-14-3-3 (1/100), anti-enolase (1/100), and anti-HSP70 (1/100); primary rabbit antibodies anti-p-P38 (1/1,000), anti-total P38 (1/1,000), anti-p-ERK1/2 (1/1,000), anti-total ERK1/2 (1/1,000), anti-p-JNK (1/1,000), and anti-total JNK (1/1,000) (all from Cell Signaling Technology, USA); and sera from *N. caninum*-infected or healthy mice (1/200) (prepared in our lab). For all secondary antibody incubations, HRP-conjugated goat anti-mouse or goat anti-rabbit antibodies (Proteintech, Wuhan, China) were used at a 1:2,000 dilution. The membranes were visualized using an ECL Western Blot Detection System (Clinx Science Instruments Co., Ltd., Shanghai, China).

### Cytokine Quantification

For the detection of inflammatory cytokines, BMDMs were seeded in 24-well culture plates at a density of 6 × 10^5^ cells/well and treated with 50 µg/ml purified *N. caninum* EVs or Pam3CSK4 (10 µg/ml) for 8, 12, and 24 h. In some experiments, the BMDMs were pretreated for 2 h with a P38 inhibitor (SB203580; 30 µM), 1 h with an ERK inhibitor (PD98059; 40 µM), 1 h with a JNK inhibitor (SP600125; 10 µM) or with PBS at 37°C before the treatment with *N. caninum* EVs. After 8, 12, or 24 h of stimulation, all supernatants were collected for ELISA assays, and Cytokine ELISA Ready-SET-Go kits (Thermo Scientific, San Diego, CA, USA) were used to determine IL-12p40, TNF-α, IFN-γ, IL-10, IL-1β, and IL-6 levels following the manufacturer’s instructions. The assays were read at 450 nm and the OD values obtained were converted to pg/ml by interpolation of the standard curve.

### Statistical Analysis

Data are analysis by one-way analysis of variance using SPSS 19.0 software (SPSS Inc., Chicago, IL, USA) and expressed as the mean ± SEM. The graphs were generated in GraphPad Prism 7.00. All experiments were performed three times with three technical replicates. Significance is shown as *P < 0.05, **P < 0.01, and ***P < 0.001.

## Results

### *N. caninum* Continuously Secretes EVs

**Figure 1:**
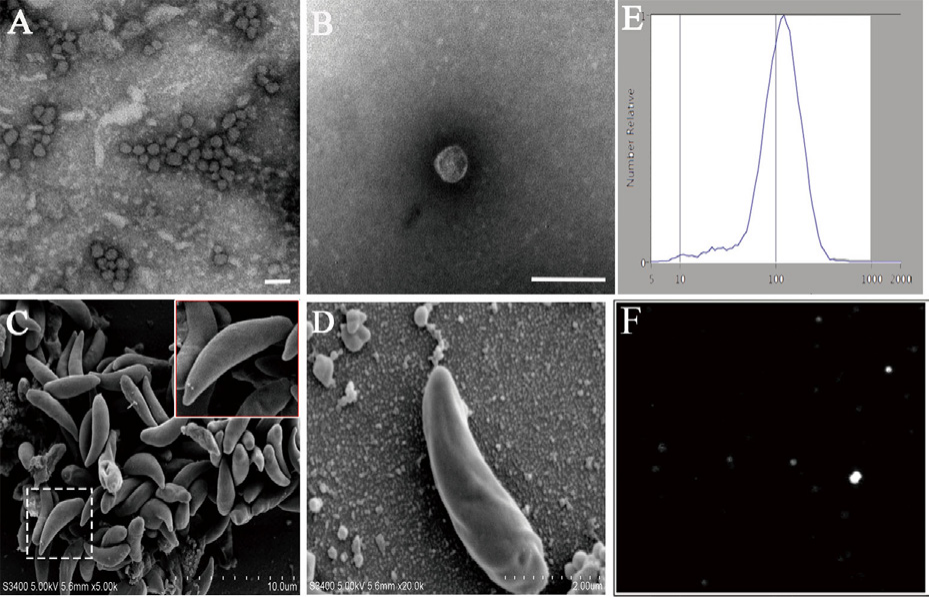
Physical characterization of *Neospora caninum* extracellular vesicles (EVs). **(A,B)** Images of the rounded or cup-shaped vesicles obtained using negative staining by TEM. **(C,D)** *N. caninum* tachyzoites were cultured in 24-well plates. After 6 h, EVs on tachyzoites surfaces were observed as shown by SEM [**(D)** shows a higher magnification of **(C)**]. **(E)** Nanosight trace of purified *N. caninum* EVs. A mean diameter of 105 nm was measured. **(F)** The concentration and diameter detection of *N. caninum* EVs using Nanosight. See also Video S1 in Supplementary Material. Scale bars: 200 nm.

**Figure 2:**
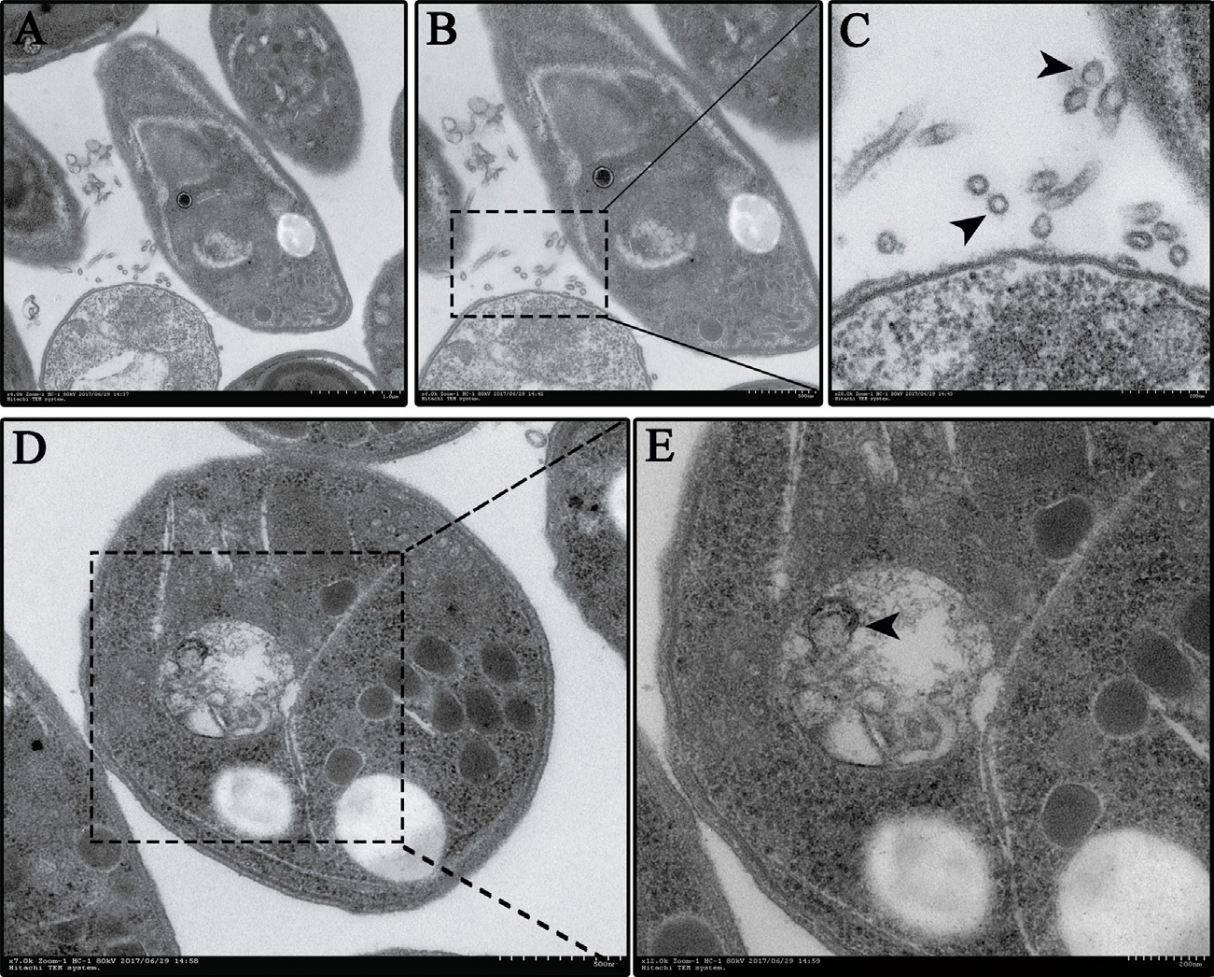
*Neospora caninum* continuously releases extracellular vesicles (EVs) to the external environment. **(A–C)** EVs on the *N. caninum* surface were observed using TEM. Panels **(B,C)** show enlargements of the section indicated by a square in panel **(A)**. **(D,E)** Multivesicular body-like structures are present in the *N. caninum* interior. Panel **(E)** shows enlargements of the sections indicated by the square in panel **(D)**.

To test whether *N. caninum* produces EVs, vesicles were isolated from parasite growth media through a series of ultracentrifugation steps, similar to that described for isolating exosomes from the parasitic protozoan *Leishmania* (24). The purified vesicles were visualized using negative staining by TEM, which showed that these vesicles were rounded or cup-shaped, limited by a membrane bilayer and approximately 50–150 nm in diameter (Figures 1A,B). The SEM of *N. caninum* tachyzoites that were cultured in 24-well plates for 6 h showed the presence of EVs on the surfaces of *N. caninum* (Figures 1C,D). To determine the sizes of EVs, we used nanoparticle tracking analysis to directly examine millions of vesicles. This analysis showed that the mean EVs diameter was 105 nm, and most were between 50 and 150 nm in size (Figures 1E,F; Video S1 in Supplementary Material). The TEM results showed that multivesicular body like structures were present in *N. caninum* (Figures 2D,E). A section of the field is presented in Figure 2, which reveals the bilayer boundary membrane of *N. caninum* EVs (Figure 2). Each panel is presented at two magnifications. However, the exact mechanism of production, interaction, and function of *N. caninum* EVs needs further study.

### Proteomic Analysis of *N. caninum* EVs

**Figure 3:**
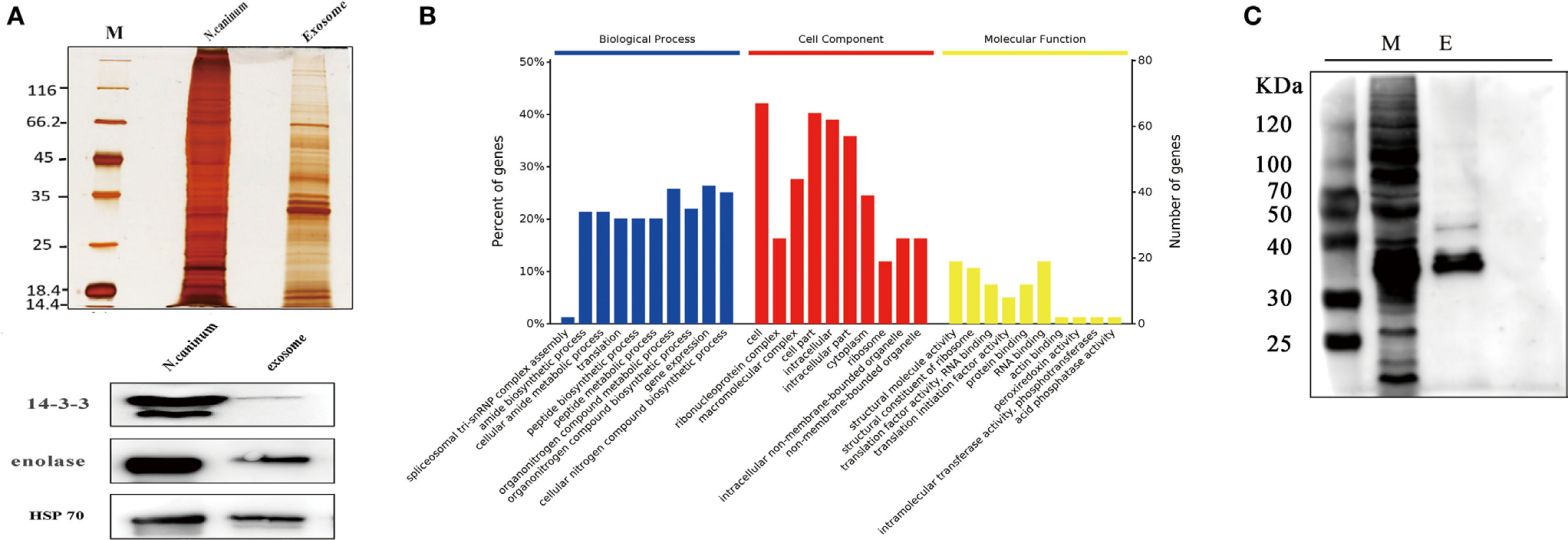
Analysis of proteins in *Neospora caninum* extracellular vesicles (EVs). **(A)** Top, silver staining of the total proteins and EVs *of N. caninum*. Bottom, western blotting analyses of 14-3-3, enolase, and HSP70 in the purified EVs. **(B)** Gene ontology annotations for all 705 proteins in the *N. caninum* EVs. **(C)** Western blot analysis of the EVs using anti-*N. caninum* mouse serum. M, total crude proteins of *N. caninum*; E, total proteins of the *N. caninum* EVs.

The proteome results shown that 705 proteins were identified in the EVs secreted by *N. caninum*, for which 65.5% (462/705) were detected with at least two unique peptides (Table S1 in Supplementary Material). Among these proteins, 556 were grouped into 97 pathways, the top five of which were Ribosome, Carbon metabolism, Spliceosome, RNA transport, and Proteasome. The pathways also included the PI3K-Akt signaling pathway, MAPK signaling pathway, NOD-like receptor signaling pathway, and toll signaling pathway (Table S2 in Supplementary Material). When we compared these results with the exosomal proteins in ExoCarta, we found extensive overlaps with other protozoan parasites, such as *Trichomonas vaginalis, Echinococcus multilocularis*, and *Leishmania* (29). Proteins involved in EV biogenesis and trafficking, such as 14-3-3, HSP70, and HSP90, were highly enriched in the purified EVs (Table 1), in accordance with the results of the identification of EVs using anti-14-3-3, anti-HSP70, and anti-enolase antibodies (Figure 3A). In addition, *N. caninum* EVs were enriched in membrane-associated parasite antigens, including surface protein P36, MIC families, and SAG families (Table 1). The percent of *N. caninum* EVs genes relating to biological process, cell component, and molecular function was showed in Figure 3B. Proteins loaded into the EVs, especially those with molecular weights of 30–50 kDa, were specifically recognized by mouse anti-*N. caninum* serum (Figure 3C), which suggested that *N. caninum* EVs are involved in inducing the host cell immune responses.

**Table 1:** Top 50 proteins enriched in *Neospora caninum* extracellular vesicles.

Proteins	Peptides	PSMs	Unique peptides	Accession
Surface protein P36	19	316	0	AAF32519.1
p29 surface antigen, partial	18	289	1	AAD39489.1
Putative surface antigen protein 1	14	192	1	AAO85715.1
Microneme protein Nc-P38	22	106	22	AAF19184.1
Unnamed protein product	23	75	23	XP_003879744.1
Tachyzoite surface protein	18	87	18	AAX38600.1
Conserved hypothetical protein	8	65	8	CBZ51310.1
Cell division control protein 48 homolog A	33	41	28	CEL67655.1
SRS domain-containing protein	17	68	6	CEL71228.1
Putative NAD-specific glutamate dehydrogenase	31	42	31	XP_003886159.1
SRS domain-containing protein	14	60	10	CBZ50604.1
SRS domain-containing protein	11	48	11	CEL70383.1
Actin, related	16	33	16	CBZ49859.1
Hypothetical protein NCLIV_032660	16	34	16	XP_003883511.1
SRS domain-containing protein	13	41	0	CBZ50603.1
KH domain-containing protein, putative	20	25	20	CEL70275.1
Conserved hypothetical protein	17	37	17	XP_003879576.1
Poly(ADP-ribose) glycohydrolase pme-3	23	28	23	CEL66156.1
SSU ribosomal protein S3P, related	10	28	10	XP_003883474.1
KH domain-containing protein, putative	22	30	22	CEL69307.1
Staphylococcal nuclease domain-containing protein 1	17	18	17	CEL65707.1
Fructose-1 6-biphosphatase, related	14	21	14	CBZ56098.1
Elongation factor 1-alpha, related	17	22	17	XP_003879714.1
Myosin, heavy polypeptide 1, skeletal muscle, adult, related	25	25	25	CEL66663.1
Subtilisin-like serine protease, partial	11	23	11	AAF04257.1
Hypothetical protein BN1204_032270	15	24	15	CEL67427.1
S15 sporozoite-expressed protein	16	21	16	CEL66440.1
Putative elongation factor 2	25	32	25	XP_003882268.1
Heat shock protein 70, related	18	21	17	CEL67598.1
Kringle domain-containing protein	19	19	19	CEL66811.1
Hypothetical protein NCLIV_039400	19	27	19	CBZ50865.1
Hypothetical protein NCLIV_047860	11	17	11	XP_003884386.1
DEAD/DEAH box helicase, putative	27	29	27	CEL64231.1
50S ribosomal protein L4P, related	14	24	14	XP_003884959.1
DEAD-box ATP-dependent RNA helicase 34, related	14	18	14	CEL70968.1
Dynamin-like protein, putative	23	24	23	CEL64534.1
Heat shock protein 70 (precursor), related	14	15	13	CBZ55124.1
Putative 2-hydroxyacid dehydrogenase SACOL2296	12	14	12	CEL65811.1
Unnamed protein product	17	18	17	CBZ55111.1
Hypothetical protein NCLIV_012890	21	22	21	XP_003881528.1
Glucose-6-phosphate dehydrogenase, putative	14	20	13	CEL64180.1

### *N. caninum* EVs Could Transfer Antigens to BMDMs

**Figure 4:**
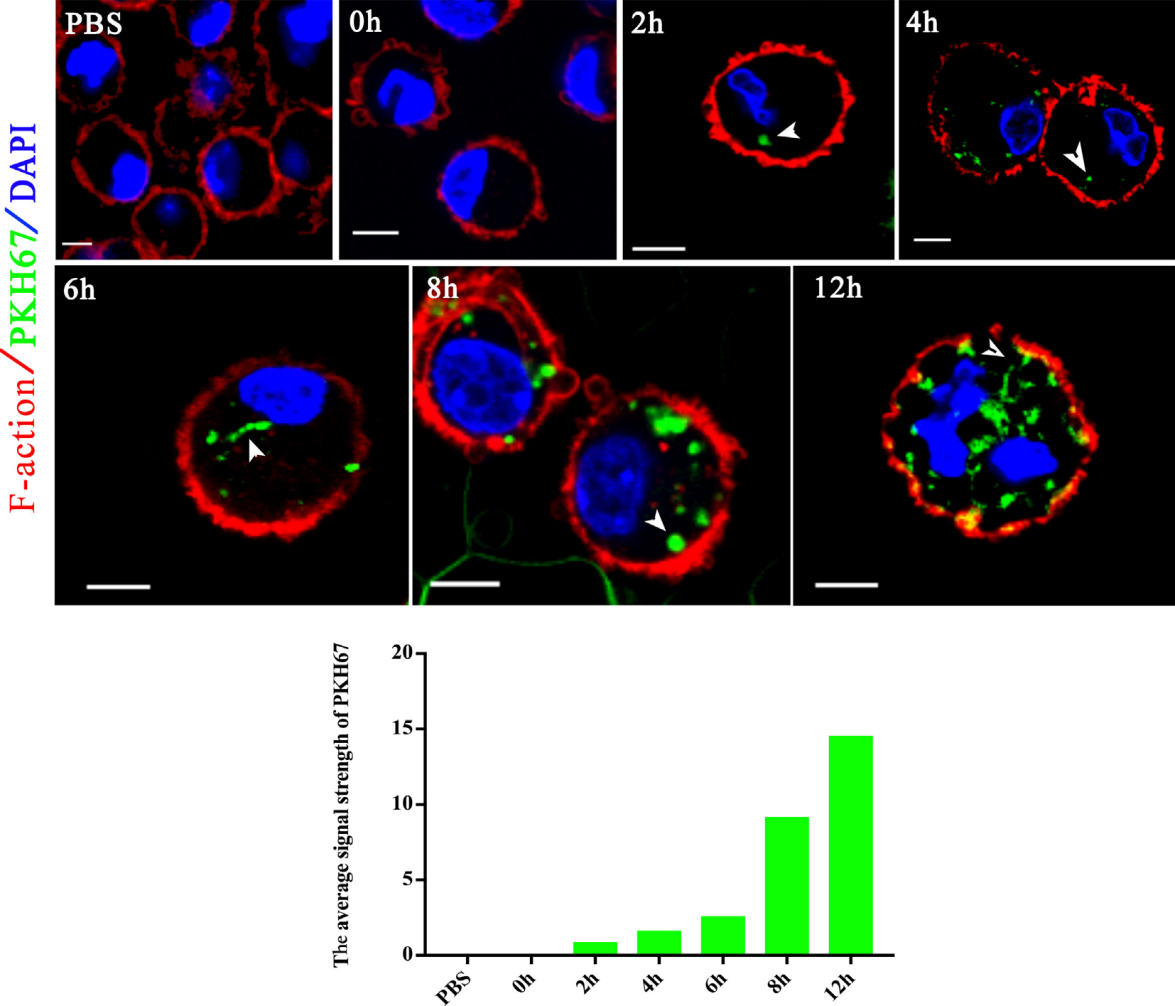
*Neospora caninum* extracellular vesicles (EVs) are released into bone marrow-derived macrophages (BMDMs). BMDMs were incubated with 50 µg/ml *N. caninum* EVs. After 0, 2, 4, 6, 8, and 12 h, the cells were processed for imaging by confocal microscopy: green, PKH67-labeled EVs; red, host F-actin; blue, nuclei. The negative control consisted of mouse BMDMs treated with PKH67-PBS. Scale bars: 5 µm.

Extracellular vesicles can transport molecules from pathogens to hosts and can transfer antigens as well as infectious agents (30). Previously, EVs were proposed to be a mechanism for the delivery of *Leishmania* molecules directly into macrophages and the regulation of the immune response (20). Hence, we hypothesized that *N. caninum* EVs can deliver *N. caninum* molecules directly into the macrophages. To examine this hypothesis, 50 µg of *N. caninum* EVs were labeled *in vitro* with the green fluorescent lipid dye PKH67 prior to exposure, and BMDMs were examined by confocal microscope at various times as shown in Figure 4. We found that as early as 2 h after *N. caninum* EVs treatment, green fluorescence was found in the cytoplasm of BMDMs, and the fluorescence intensity increased steadily at time points later than 2 h. These data indicate that *N. caninum* EVs were internalized by the exposed host cells.

**Figure 5:**
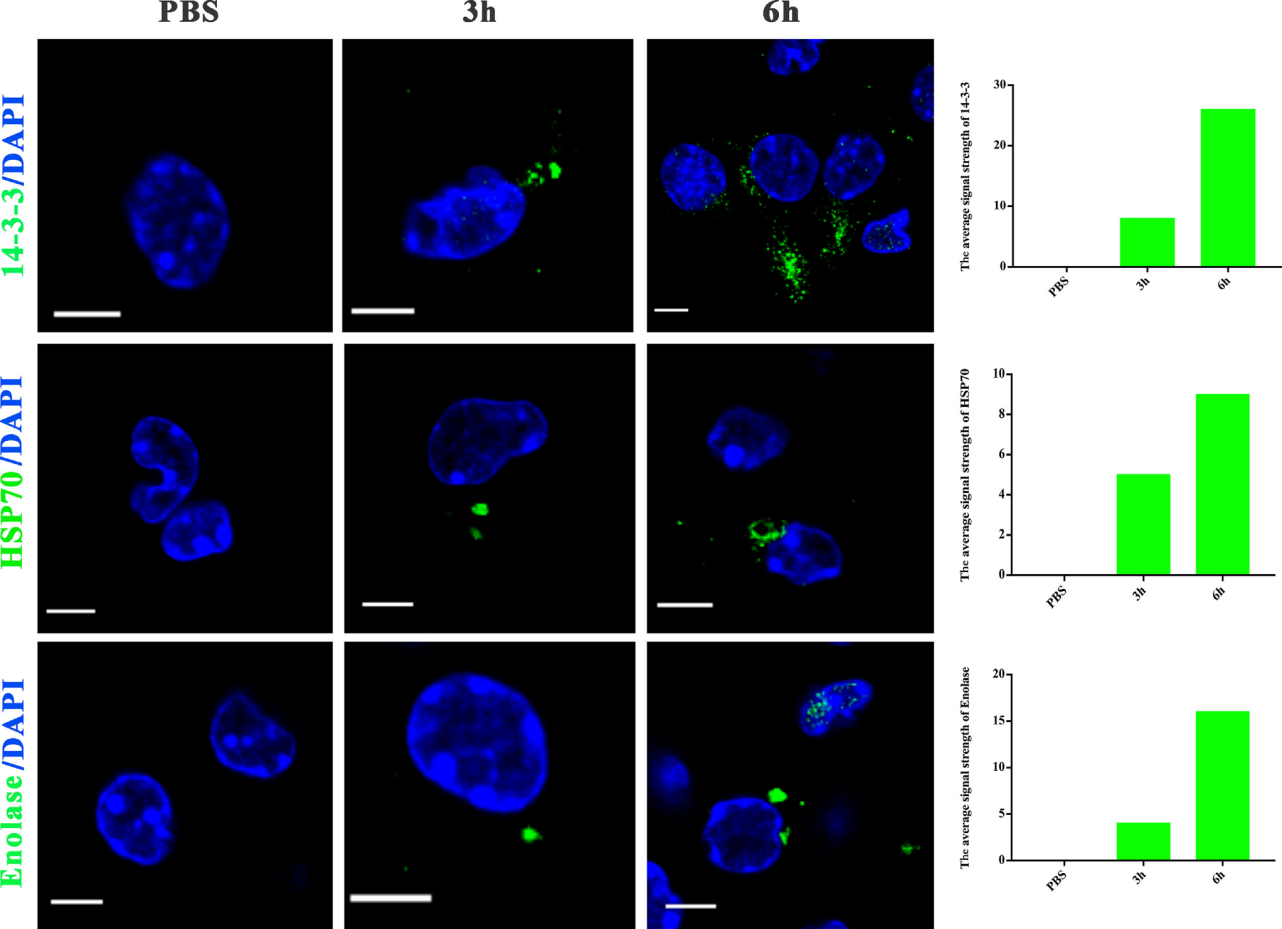
*Neospora caninum* extracellular vesicles (EVs) can deliver proteins to bone marrow-derived macrophages (BMDMs). BMDMs were incubated for 3 or 6 h with PBS or 50 µg/ml *N. caninum* EVs, then exposed to *N. caninum*-specific antibodies against 14-3-3, HSP70, or enolase and an Alexa Fluor-conjugated secondary antibody for confocal microscopy. Green: the 14-3-3, HSP70, and enolase proteins. Blue: nuclei. Scale bar: 5 µm.

To further examine whether proteins in *N. caninum* EVs are delivered to host cells, we utilized *N. caninum*-specific antibodies against 14-3-3, HSP70, and enolase for confocal microscopy. As shown in Figure 5, after *N. caninum* EVs treatment, fluorescence was observed specifically in the cytoplasm of infected BMDMs in a time-dependent manner, but no fluorescence was observed in the PBS-treated group. These results provide strong evidence that *N. caninum* EVs can deliver their contents to BMDMs.

### *N. caninum* EVs Induce Cytokines Expression in BMDMs

**Figure 6:**
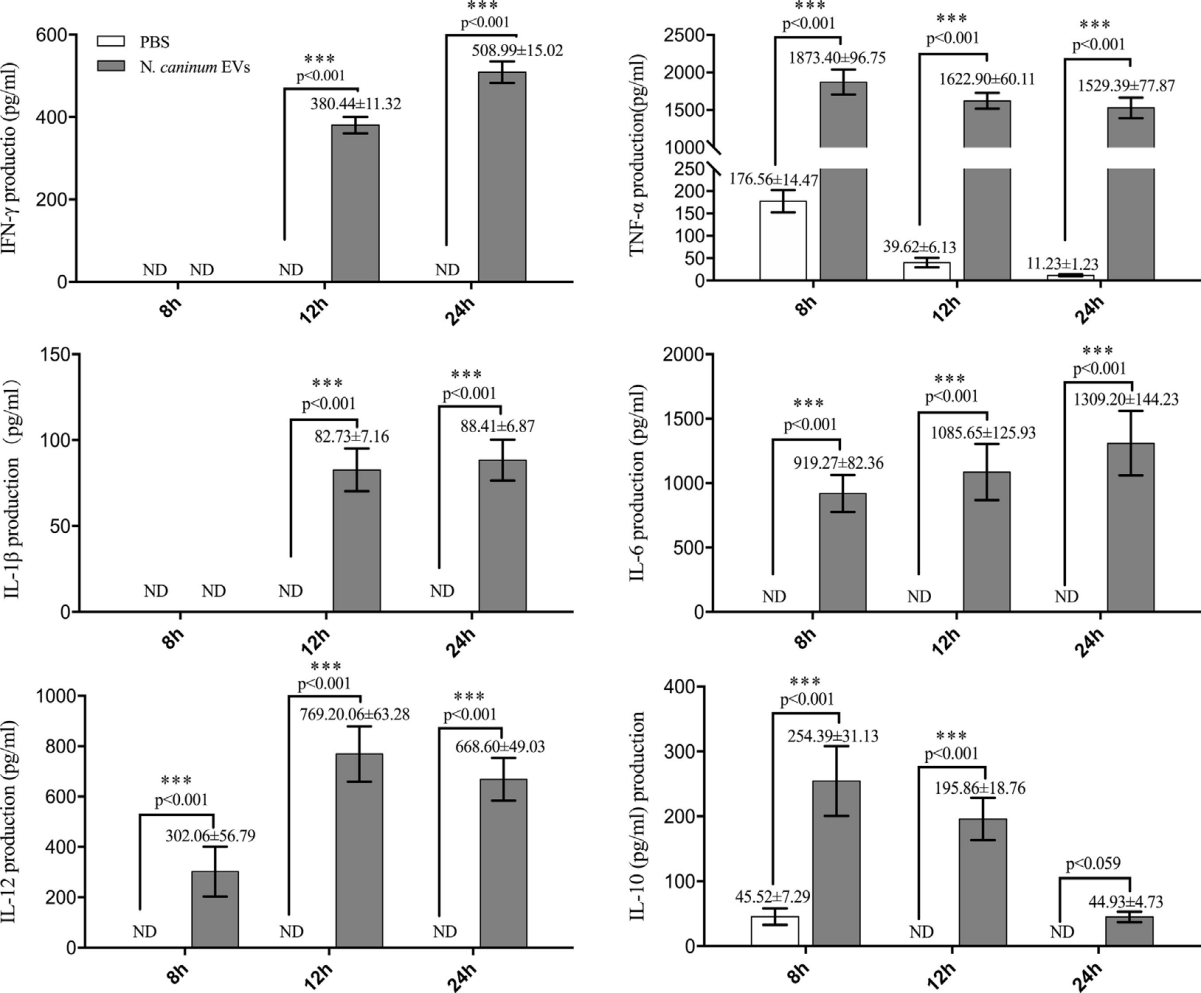
*Neospora caninum* extracellular vesicles (EVs) can induce cytokines expression in bone marrow-derived macrophages (BMDMs). BMDMs were incubated with either PBS or 50 µg/ml *N. caninum* EVs for 8, 12, or 24 h, and the supernatants were removed and assayed for IL-12p40, TNF-α, IL-1β, IL-6, IFN-γ, and IL-10 cytokines using ELISA assays. The data are representative of three independent experiments and are presented as the mean ± SEM. ***P < 0.001 for the *N. caninum* EVs group versus the PBS group.

LAL assays showed that the concentration of endotoxin in *N. caninum* EVs, NLA, and ESA were 0.033, 0.176, or 0.042 EU/ml, respectively, which could not induce TLR2/TLR4 activation or the production of IL-8 or TNF-α (31). The immune response to *N. caninum* infection is predominantly the T helper 1 (Th1)-type, in which IL-12, IFN-γ, and NO are sequentially produced by the cells of the immune system (32). In this study, we hypothesized that *N. caninum* EVs may modulate the cytokines expression of BMDMs. Thus, we examined the inflammatory cytokines IL-12p40, TNF-α, IL-1β, IL-6, IFN-γ, and IL-10, which are secreted by BMDMs in response to *N. caninum* EVs after 8, 12, and 24 h. The levels of IL-12p40, TNF-α, IL-1β, IL-6, IFN-γ, and IL-10 were dramatically increased in the BMDMs that were treated with *N. caninum* EVs compared with those in the PBS group (Figure 6).

### *N. caninum* EVs Increase Cytokine Secretion in BMDMs by Activating TLR2 Signaling Pathway

**Figure 7:**
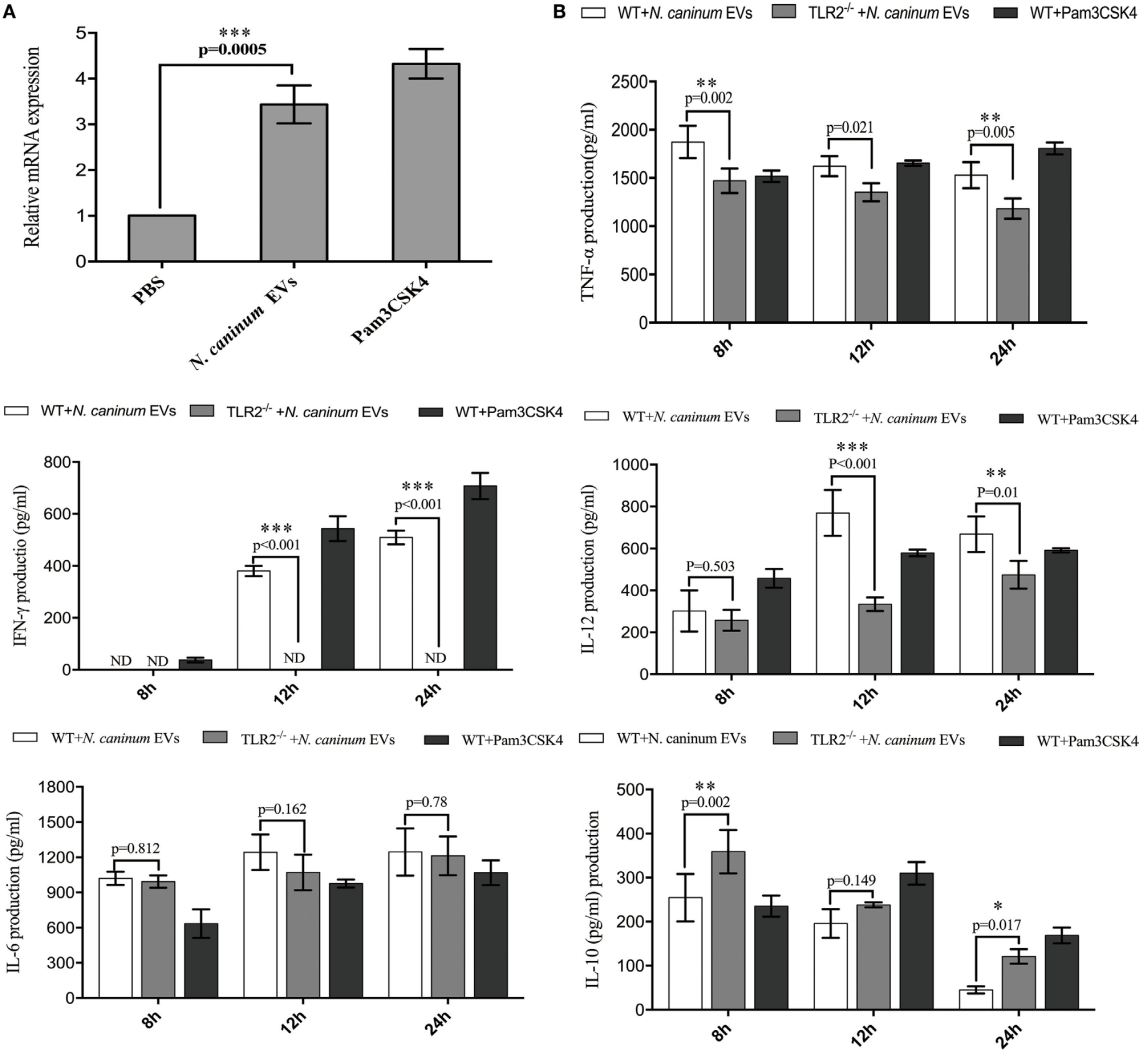
*Neospora caninum* extracellular vesicles (EVs) induce cytokine secretion in bone marrow-derived macrophages (BMDMs) by activating toll-like receptor 2 (TLR2) signaling pathway. **(A)** RT-PCR analysis of the relative mRNA level of TLR2 in total RNA isolated from 3 × 10^6^ BMDMs incubated with PBS, 10 µg/ml Pam3CSK4 or 50 µg/ml *N. caninum* EVs. The mRNA levels were normalized to those of GAPDH. **(B)** TLR2^−/−^ and wild-type (WT) mouse BMDMs were incubated with 50 µg/ml *N. caninum* EVs for 8, 12, and 24 h, and the supernatants were removed and assayed for IL-12p40, TNF-α, IL-1β, IL-6, IFN-γ, and IL-10 using ELISA assays. The data are representative of three independent experiments and are presented as the mean ± SEM. *P < 0.05; **P < 0.01; ***P < 0.001 for the TLR2^−/−^ group versus the WT group.

Toll-like receptor 2 is the innate receptor with the broadest range of recognition, being able to identify diverse pathogen compounds (33, 34). Previous studies showed that live *N. caninum* tachyzoites can induce TLR2 upregulation and regulate the production of inflammatory cytokines to control the infection of *N. caninum*. However, which PAMPs are involved in this process has not yet been fully elucidated, and the mechanisms by which antigens are presented to BMDMs remain unclear. In this study, we examined whether *N. caninum* EVs affect the production of inflammatory cytokines through TLR2. As we expected, when BMDMs were incubated with *N. caninum* EVs for 12 h, a significant upregulation of TLR2 resulted compared with the negative control cells (Figure 7A). Next, to directly examine the role of TLR2 in cytokine production during treatment with *N. caninum* EVs, the culture supernatants of BMDMs generated from WT and TLR2^−/−^ mice were examined after incubation with or without *N. caninum* EVs for 8, 12, and 24 h. We found that the BMDMs from TLR2^−/−^ exposed to *N. caninum* EVs showed extremely downregulated secretion of IL-12p40, TNF-α, and IFN-γ compared with the BMDMs from the WT mice, although the change in IL-6 was not significant. We also observed upregulation of IL-10 (Figure 7B).

### *N. caninum* EVs Induce Cytokine Expression by Activating MAPK Pathways

**Figure 8:**
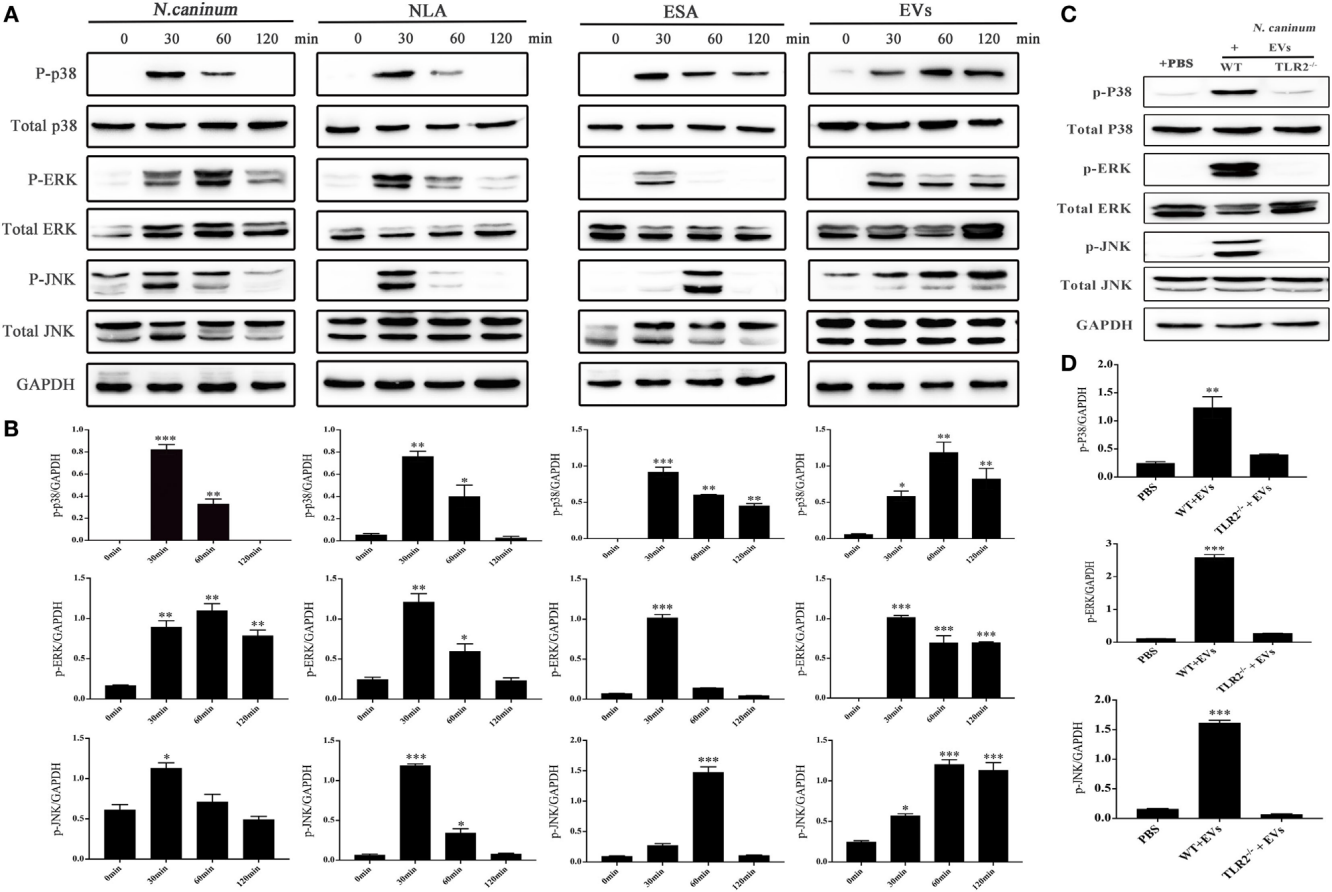
*Neospora caninum* extracellular vesicles (EVs) induce the phosphorylation of P38, ERK, and JNK *via* toll-like receptor 2 (TLR2). **(A)** Wild-type (WT) mouse bone marrow-derived macrophages (BMDMs) (3 × 10^6^ cells) were stimulated with *N. caninum* tachyzoites (MOI = 3:1, parasite:cell) or 50 µg/ml *N. caninum*-derived polypeptides [*Neospora caninum* lysate antigen (NLA), excretory secretory antigen (ESA), or EVs] for the indicated times (0, 30, 60, and 120 min) as indicated, then the phosphorylation levels of P38, ERK, and JNK were determined by immunoblot analysis. **(B)** Relative levels of the signals from the western blot in panel **(A)**. **(C)** WT and TLR2^−/−^ mouse BMDMs (3 × 10^6^ cells) were stimulated with 50 µg/ml *N. caninum* EVs for 60 min, and then the p-P38, p-ERK, and p-JNK expression were determined by immunoblot analysis. **(D)** Relative levels of the signals from the western blot in panel **(C)**. The phosphorylated forms of these proteins were clearly observed at 30 min after treatment. The data are expressed as the mean ± SEM from three separate experiments, *P < 0.05; **P < 0.01; ***P < 0.001 for *N. caninum* EVs group versus the PBS groups.

To determine the effect of *N. caninum* EVs on MAPK activation in BMDMs, the phosphorylation of the major MAPK signaling components was examined using western blot and phospho-specific antibodies. The results showed that *N. caninum* EVs could induced obvious phosphorylation of P38, ERK, and JNK after treatment for 30 min. The phosphorylation level of P38 and ERK gradually reduced within the indicated time, but p-JNK did not decreased at 120 min (Figures 8A,B). In the context of these findings, we suggested that *N. caninum* EVs included some antigenic components that can predominantly activated MAPK after stimulation on BMDMs. Furthermore, we also evaluated the effect of *N. caninum* tachyzoites (MOI = 3:1, parasite:cell) and *N. caninum*-derived polypeptides (NLA, ESA) on MAPK activation in BMDMs. The results showed that all of these proteins could induce the phosphorylation of p38, ERK, and JNK after treatment for 30 or 60 min (Figures 8A,B). Taken together, these data suggested that *N. caninum* activates the MAPK signaling pathway in BMDMs, probably through a component of its excreted/secreted antigens, especially proteins in *N. caninum* EVs.

To explore whether *N. caninum* EVs induced the phosphorylation of the major MAPK signaling components through TLR2, we treated BMDMs (WT and TLR2^−/−^) with PBS or *N. caninum* EVs for 60 min at 37°C. The phosphorylation levels of p38, ERK, and JNK were significantly reduced in the TLR2^−/−^ cells, which demonstrated that *N. caninum* EVs induced phosphorylation of P38, ERK, and JNK *via* TLR2 (Figures 8C,D).

**Figure 9:**
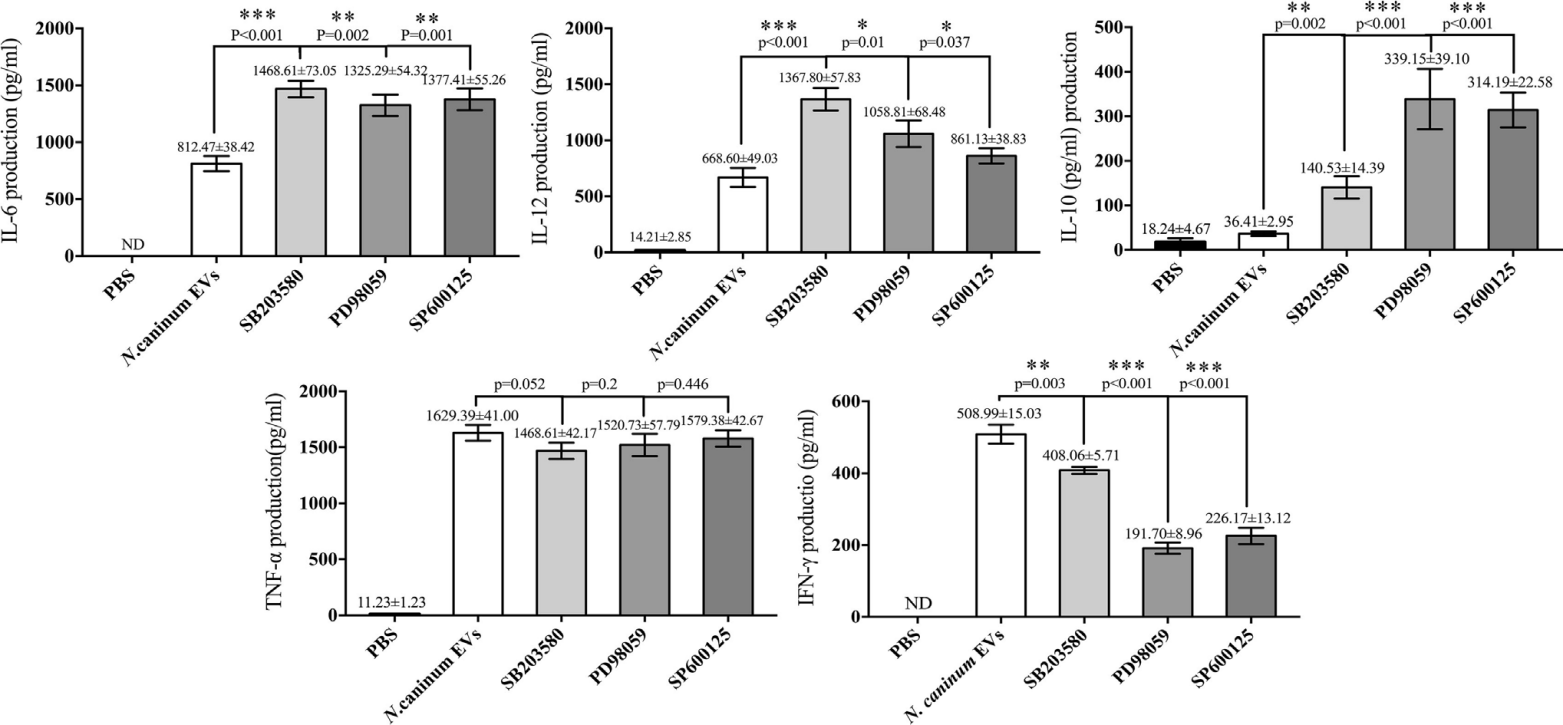
Treatment with p38, ERK, and JNK inhibitors can selectively enhance the cytokine expression. Wild-type mouse bone marrow-derived macrophages (1 × 10^6^ cell/ml) were pretreated for 2 h with a P38 inhibitor (SB203580; 30 µM), for 1 h with an ERK inhibitor (PD98059; 40 µM), or for 1 h with a JNK inhibitor (SP600125; 10 µM) before stimulation with *Neospora caninum* extracellular vesicles (EVs). After 24 h, the supernatants were removed and assayed for IL-12p40, TNF-α, IL-1β, IL-6, IFN-γ, and IL-10 using ELISA assays. The data are representative of three independent experiments and are presented as the mean ± SEM. *P < 0.05; **P < 0.01; ***P < 0.001 for the inhibitor-treated group versus the EVs group.

We further verified the roles of P38, ERK, and JNK signaling pathways in the regulation of TNF-α, IFN-γ, IL-6, IL-12 p40, and IL-10 expression in *N. caninum* EVs-stimulated BMDMs. For cells in which P38, ERK, and JNK activities were blocked with their respective inhibitors SB203580, PD98059, and SP600125, the ELISA assays showed that treatment of BMDMs with the inhibitors could significantly increase the production of IL-6, IL-12p40, and IL-10. By contrast, the secretion of IFN-γ was dramatically decreased, and the level of TNF-α was not significantly different from that in BMDMs treated only with *N. caninum* EVs (Figure 9).

## Discussion

Many parasites use multiplex mechanisms to survive in the hosts. Previous research has suggested that many parasites deploy EVs for biologically active effector molecules delivery as a means of cell–cell communication, especially among immune cells (13, 35). In our study, we first isolated EVs from the NC-1 strain of *N. caninum* and described the composition and characteristics of these EVs. Furthermore, our results indicated that these EVs could modulate the cytokines expression in BMDMs through TLR2 and MAPK signaling pathway since they delivered their contents to BMDMs.

We show herein that *N. caninum* EVs are spherical vesicles with 50–150 nm in diameter, the same characteristics as classical exosome-like EVs (36). Proteomics results showed that *N. caninum* EVs were enriched in secreted and membrane-associated proteins, including the 14-3-3 and HSP70 and HSP90, which have been extensively used for exosome definition in *E. multilocularis* (37) and *Leishmania* (29). Studies have shown that EVs could use direct membrane fusion or receptor-mediated mechanism to transfer genetic materials, proteins, and lipids to cells (14, 38). In this study, we used the green fluorescent lipid dye PKH67 to label *N. caninum* EVs *in vitro* prior to treating BMDMs and found that the green fluorescence moved into the cytoplasm and that the fluorescence intensity grew stronger with time. Subsequent experiments, which employed *N. caninum*-specific antibodies against 14-3-3, HSP70, and enolase, also confirmed that *N. caninum* EVs could be internalized in and delivered their contents to BMDMs. In summary, these results supported that *N. caninum* can use EVs to deliver effector molecules to host cells.

Recently, many studies have shown that EVs are a strategy that is used by parasites to beneficial changes in the host environment and to ensure a successful infection or to activate the innate immune response to control the infection (39). Exosomes from *T. gondii* infected macrophages could trigger a pro-inflammatory response both *in vitro* and *in vivo* (40). Recently, parasite antigen-containing exosomes isolated from the sera of the infected chickens were shown to induce protection against *Eimeria tenella* (41). Similar to these findings, we observed that *N. caninum* EVs proteins reacted with mouse anti-*N. caninum* serum, and proteins with molecular weights of 30–50 kDa were specifically recognized, which demonstrated that *N. caninum* EVs were likely to be involved in host immune responses. The Th1 immune response and, in particular, the production of the pro-inflammatory cytokines IL-12p40, IFN-γ, and TNF-α is considered to be a keystone of the protection against *N. caninum* infection (42). In our study, *N. caninum* EVs were found to induce IL-12p40, TNF-α, IL-1β, IL-6, and IFN-γ responses after stimulation of BMDMs for 8, 12, and 24 h, whereas IL-10 was increased at 8 and 12 h but subsided at 24 h. The high production of IL-10 in BMDMs during *N. caninum* EVs treatment was unexpected. Since IL-10 is a known potent anti-inflammatory cytokine involved in immune suppression during intracellular parasite infection (43), the elevated production of IL-10 from exposure to *N. caninum* EVs suggested a potential role for these vesicles in promoting sustained survival of parasites within the host cells. These results demonstrated that *N. caninum* may use EVs to manipulate the host defense responses similar to *Leishmania species* and *T. cruzi* (44, 45).

We now know that innate immune receptors, including TLRs and NLRs, are responsible for triggering the inflammatory environment against pathogens and initiating the immune protective response (46). Of all TLRs described, TLR2 recognizes the widest range of PAMPs, and the TLR2/MyD88 pathway is essential for DCs to efficiently prime adaptive immune system against *N. caninum*. This has the consequence of reducing the parasite burden during the acute and chronic phases of infection (33). However, which PAMPs participate in this pathway has not yet been fully elucidated. In our study, we found that exposure of the BMDMs from TLR2^−/−^ mice to *N. caninum* EVs resulted in an extreme downregulation of the secretion of IL-12p40, TNF-α, and IFN-γ but not IL-6. By contrast, IL-10 was upregulated. Studies have shown that *N. caninum* induces a TLR2-dependent production of the pro-inflammatory cytokines IL-12p40 and IFN-γ. However, the production of anti-inflammatory IL-10 during infection has not been clearly demonstrated. *Leishmania* exosomes could selectively induce the secretion of IL-8, IL-10, and a Th2 response in DCs. Exosomes released from the DCs pulsed with *T. gondii* proteins could stimulate TNF-α production as a protective immune response against acute and chronic *T. gondii* infection when adoptively transferred to mice (47). Unexpectedly, there was no difference in the production of IL-6 between the WT and TLR2^−/−^ BMDMs exposed to *N. caninum* EVs, but this result was consistent with Antonella Cano’s findings, in which the production was not diminished in TLR2^−/−^ macrophages infected with *Acanthamoeba castellanii* (48). IL-6 is a pro-inflammatory cytokine in the acute innate response after parasite infection and has been implicated as necessary for anti-*T. gondii* and *N. caninum* immunity (49, 50). Previous research has shown that TLR4 mediated the production of the inflammatory cytokine IL-6 and strongly promoted Th17 cell differentiation (51). A recent study showed that changing the micro milieu in inflammation also changes the function of IL-6 to become more anti-inflammatory (52). The signaling cassette that controls the activity of IL-6 in response to *N. caninum* EVs is complicated, but whether TLR4 is involved remains to be further studied. Together with the above results, our study showed that TLR2 could be distinctly activated during *N. caninum* EVs stimulation in BMDMs, and some PAMPs that can trigger TLR2 were present in *N. caninum* EVs. Furthermore, *N. caninum* EVs could modulate the cytokines IL-12p40, TNF-α, IL-10, and IFN-γ of BMDMs in a manner dependent on TLR2.

To address the mechanism of the *N. caninum* EVs-mediated immune response, we evaluated the possible participation of MAPK pathways. We observed that *N. caninum* EVs could induce the phosphorylation of major MAPK pathway proteins (P38, ERK, and JNK) and significantly increased production of IL-6, IL-12, and IL-10 when BMDMs were separately pretreated with P38, ERK, and JNK inhibitors. In conclusion, the secretion of inflammatory cytokines in BMDMs induced by *N. caninum* EVs was regulated through the MAPK signaling pathway.

We demonstrated here that EVs released by *N. caninum* could modulate the cytokines expression of BMDMs by triggering TLR2 and MAPK signaling pathways, but the exact mechanism was not yet fully understood. In addition, the role of EVs in regulating the immune response *in vivo* is presently unknown. Our current knowledge of *N. caninum* EVs biogenesis did not allow for specific inhibition of the production or release of EVs. Direct evaluation of the importance of EVs during an *in vivo* infection is therefore currently not possible. In summary, our results suggested that *N. caninum* EVs are actively involved in the host–parasite interactions during the *N. caninum* infection. It will be important to elucidate the biological activities of these EVs *in vivo* in future studies.

## Ethics Statement

All animal experimental procedures were performed in strict accordance with the Animal Welfare and Research Ethics Committee at Jilin University (IACUC Permit Number: 20160612).

## Author Contributions

SL, PG, XiZ, and JL drafted the main manuscript and performed the data analysis; SL, LT, CZ, and XuZ planned and performed the experiments; SL, XW, XL, PG, and JL were responsible for the experimental design; and JY, ZY, JL, and XiZ were responsible for guiding and supporting the experiments and revising the manuscript. All authors read and approved the final manuscript.

## Conflict of Interest Statement

The authors declare that the research was conducted in the absence of any commercial or financial relationships that could be construed as a potential conflict of interest.

## Supplementary Material

Video S1 ZetaView nanoparticle tracking analysis (AVI).

Table S1 Protein repertoire identified in *Neospora caninum* extracellular vesicles (EVs) (XLSX).

Table S2 Pathways identified in which *Neospora caninum* extracellular vesicles (EVs) proteins participated (XLSX).
